# Using Latent Class Analysis to Identify Health Lifestyle Profiles and Their Association with Suicidality among Adolescents in Benin

**DOI:** 10.3390/ijerph18168602

**Published:** 2021-08-15

**Authors:** Fanny Hoogstoel, Lucresse Corine Fassinou, Sékou Samadoulougou, Céline Mahieu, Yves Coppieters, Fati Kirakoya-Samadoulougou

**Affiliations:** 1 Centre de Recherche en Epidémiologie, Biostatistique et Recherche Clinique, Ecole de Santé Publique, Université libre de Bruxelles (ULB), Route de Lennik, 808, 1070 Brussels, Belgium; fanny.hoogstoel@ulb.be (F.H.); corinefas123@gmail.com (L.C.F.); yves.coppieters@ulb.be (Y.C.); 2 Evaluation Platform on Obesity Prevention, Quebec Heart and Lung Institute, Quebec City, QC G1V 4G5, Canada; ouindpanga-sekou.samadoulougou.1@ulaval.ca; 3 Centre for Research on Planning and Development (CRAD), Laval University, Quebec City, QC G1V 0A6, Canada; 4 Centre de Recherche Interdisciplinaire en Approches Sociales de la Santé, Ecole de Santé Publique, Université libre de Bruxelles (ULB), Route de Lennik, 808, 1070 Brussels, Belgium; celine.j.mahieu@ulb.be; 5 Centre de Recherche Politiques et Systèmes de Santé, Ecole de Santé Publique, Université libre de Bruxelles (ULB), Route de Lennik, 808, 1070 Brussels, Belgium

**Keywords:** suicidality, profiles, latent class analysis, adolescents, Benin

## Abstract

Youth suicidality is considerably prevalent in low- and middle-income countries, including Benin. Factors such as psychosocial distress, socio-environmental factors, and health risk behaviors are associated with suicidality. However, little is known about how these factors co-occur in these countries. An analysis of these factors taken together would help to identify the profiles most at risk and better target prevention policies. Our study aimed to identify profiles related to these factors and their association with suicidality among adolescents in Benin. Data from the 2016 Global School-Based Student Health Survey were used, and factors related to lifestyle (tobacco and alcohol consumption and physical activity), physical violence, parental support, and psychological distress were studied. Latent class analysis was used to identify the profiles, and a modified Poisson regression with generalized estimating equations, adjusted for sociodemographic characteristics, was performed to assess the association between these profiles and suicidality. The survey results show that globally, 13.8% of the adolescents (n = 2536) aged 11 to 18 had thought about suicide, 15.6% had planned suicide, and 15.6% had attempted suicide. Four profiles were identified: a low-risk group, one with psychological distress problems, a group with violence problems, and one with alcohol, tobacco, and violence problems. The risk of suicidality, in terms of ideation, planning, or attempting, was higher for adolescents in Profiles 2, 3, and 4 than those in Profile 1 (*p* < 0.05). Adolescents in Profile 2 were particularly affected by this increased risk (prevalence ratio (PR) for ideation = 1.13, 95% CI = 1.03–1.23; PR for planning = 1.12, 95% CI = 1.04–1.22; PR for attempting = 1.09, 95% CI = 1.01–1.17). This study highlights the typical profiles that may be linked with suicidality among adolescents in Benin. A holistic consideration of these factors could help in planning better preventive measures to reduce suicidality among adolescents in Benin.

## 1. Introduction

One of the indicators for monitoring good health and well-being, the third objective of the Sustainable Development Goals (SDGs) established by the member states of the United Nations, is the suicide mortality rate [1]. Suicide is defined as an act of fatal self-harm with some evidence of intent to die [2]. It is a real public health problem, which is responsible for nearly 800,000 deaths every year worldwide [3]. Suicide mortality accounts for nearly 1.4% of deaths worldwide, with a high prevalence in low- and middle-income countries, which account for about three-quarters of global suicides [3,4]. Youth are among the most affected by suicide, which is now the second leading cause of death among 15-to-29-year-olds worldwide [4]. A systematic review conducted in 2020 in 18 countries in sub-Saharan Africa among adolescents aged 10 to 25 years showed that the median prevalence of suicide was 16.9%, with a predominance in the West African region, where the median prevalence was found to be 24.3% [5]. Therefore, identifying risk factors linked to suicide or suicidal tendencies is a public health imperative, particularly among adolescents in low- and middle-income countries.

Suicidal behaviors are complex and result from the interaction of psychological, environmental, and/or genetic risk factors [6]. These behaviors include a set of behaviors ranging from having suicidal ideation to planning, attempting, and committing suicide [4]. These behaviors can be influenced by many risk factors, such as health and social system factors, community and relationship factors, and individual risk factors [4,7]. Unhealthy lifestyle behaviors can impact mental health and suicidal behavior by influencing emotions and judgment [8]; factors that increase the risk of suicide are sedentary behavior, underweight, obesity, cigarette smoking, alcohol abuse, poor mental health, and severe psychiatric disorders [8]. Having experiences with bullying and physical violence can also be associated with suicidality [9]. In fact, sedentary behavior, weight issues, and a lack of social support contribute to social isolation, limiting the development of social relationships, which increases the risk of developing mental health problems and suicidal ideation [8].

In sub-Saharan Africa in 2019, seven deaths per 100,000 population were reported to be due to suicide [10]. More specifically, a study published in Liberia in 2020 showed that in 2017, 26.8% of adolescents reported having had suicidal ideation in the 12 months preceding the survey, 36.5% had planned suicide, and 33.7% had attempted suicide [11]. In the same way, a study published in Ghana in 2017, showed for example that in 2012, the prevalence of suicidal behavior was 18.2% for suicidal ideation, 22.5% for planning, and 22.2% for attempting [12].

In Benin, a middle-income country [13], the situation is also quite pronounced. A study published in 2014 showed that 17.5% of adolescents had planned suicide and 5.7% had suicidal ideation; furthermore, 28.3% of adolescents had attempted suicide in the year preceding the survey and 13.5% had made multiple attempts [14].

It is imperative to identify the behavioral factors associated with suicide in order to better identify the different suicidal behaviors among adolescents and implement effective prevention policies. As suicide rates can be high, concerns about suicide have taken on the appearance of a “moral panic” in some countries [15]. Therefore, it is urgent to resolve this public health problem to avoid this situation in other places. Unfortunately, very few studies have focused on identifying the risk factors for suicide among adolescents in Benin, and among those that have, the analyses have focused on identifying factors independently of each other [14]. An analysis of these factors taken together would help to identify the profiles most at risk and better target prevention policies to significantly reduce the incidence of suicide among adolescents in Benin. An increasingly popular method for identifying profiles is latent class analysis (LCA). LCA aims to find heterogeneity within the population by analyzing individual patterns of behavior, such as mental health indicators, and finding common types, called classes or profiles [16]. Then, each individual is probabilistically assigned to a class, resulting in subgroups of individuals that are the most similar to each other and the most distinct from others [16].

Therefore, our study aimed to assess the prevalence of suicidality, identify the different behavioral profiles related to lifestyle, and determine their association with suicidality among adolescents in Benin using latent class analysis.

## 2. Materials and Methods

### 2.1. Context and Data Sources

The data used in this study were collected in the Republic of Benin as part of the Global School-Based Student Health Survey (GSHS) developed in collaboration with the World Health Organization (WHO) and the US Centers for Disease Control (CDC). The GSHS is a cross-sectional national survey conducted in WHO member countries that are interested in assessing lifestyle-related behavioral factors among schooled adolescents. Data are collected through a self-administered questionnaire, available on the WHO website [17].

The objective of this survey is to provide accurate data on health-related behaviors and protective factors for students in order to (1) help countries to develop priorities, establish programs, and promote resources for school and youth health programs and policies; (2) enable international organizations, countries, and other entities to make comparisons between countries regarding the prevalence of health behaviors and protective factors; and (3) to establish trends of the prevalence of health behaviors and protective factors by country in order to assess school health and promote the health of young people [18]. The GSHS survey was approved by the Ministry of Health in Benin. Participation was voluntary, and all the participants and their parents or guardians gave their consent.

### 2.2. Sampling

The sampling was carried out with the technical support of the CDC in Atlanta, using a random sampling technique of two degrees with a probability proportional to the size of school enrolment and class size. The first level involved selecting 40 public and private secondary schools out of a total of 1494. At the second level, a census of all classes was carried out in each selected school. The choice of classes to be studied was made at random from a list of random numbers, which were pre-established and made available by the CDC and the WHO. This list of figures varied from school to school. The number of classes to be studied per school was proportional to the total number of classes available at the selected schools. Finally, all adolescents in the eligible classes were included in the study [19]. The GSHS survey was approved by the Ministry of Health, Benin. Participation was voluntary, and all adolescents and their parents or guardians had given their consent.

A total of 2536 students from grade 6 to “terminale” were included in the study [20], with a school response rate of 100%, a student response rate of 78%, and an overall response rate of 78% [20]. Incomplete cases for this analysis represented 12.6% (319 individuals) of the adolescents.

### 2.3. Measures

The questions used in the GHSH were developed jointly by the WHO and the CDC. The variables considered in this study were chosen based on a literature review [8,11,14,21,22]. As our study was related to suicidality, the dependent variable was suicidal ideation, planning, and attempts. Factors such as psychosocial distress, socio-environmental factors, health risk behaviors, and sociodemographic characteristics were used as covariates [21]. Table 1 summarizes how the factors were measured in our study.

#### Suicidality

In our study, the term suicidality embraces suicidal thoughts, plans, and suicide attempts. In order to assess this suicidality, the GSHS survey includes a question related to attempted suicide and two questions related to suicidal ideation and planning. The last two were, respectively, “During the past 12 months, did you ever seriously consider committing suicide?” and “During the past 12 months, did you make a plan about how you would attempt suicide?”. The answers were coded “yes” and “no”. With regard to attempted suicide, the question was, “During the past 12 months, how many times did you actually attempt suicide?”. We recorded the responses as follows: “no suicide attempt” and “at least 1 suicide attempt”.

### 2.4. Statistical Analysis

Data analysis was performed with R version 4.0.3. The sampling method and sampling weight were used to generalize the results at the population level. Taking into account this weight and the sampling procedure (with the primary sampling unit (PSU) and strata variable), the proportion of each indicator was calculated using the “survey” package version 4.0.

Multiple imputations were performed on adolescents with one missing value among the eight variables studied, while those with at least two missing values were excluded (21 individuals, 0.8% of the total population). The predictive mean matching (PMM) method was applied using the “mice” package in R version 3.13.02.3.1.

To identify and describe risk behavior profiles, latent class analysis (LCA) was conducted. LCA is a measurement model that uses categorical variables to identify homogeneous, mutually exclusive, and exhaustive latent classes [23]. To perform this analysis and obtain profiles, the following factors were used: anxiety, loneliness, physical attack, physical fight, parental support, tobacco and alcohol consumption, and physical activity.

Initially, the optimal number of classes adapted to the data was determined. An exploratory approach was used, starting from a 2-class model, and the analysis was performed several times in a row by increasing the number of classes and replicating each model 10 times for greater accuracy. Second, model fit indices were used to evaluate the best model: the Akaike information criterion (*AIC*), Bayesian information criterion (*BIC*), sample-adjusted Bayesian information criterion (*SABIC*), and consistent Akaike information criterion (*CAIC*). A low value for any of these criteria indicated a better model [24]. The adjusted likelihood ratio and entropy were also used to define the best model. Indeed, the likelihood ratio provided a *p*-value that allowed us to determine whether one model was statistically better than another. Entropy, on the other hand, indicates how precisely the model defines the classes [24]. The “poLCA” package version 1.4.1 in R was used for latent class analysis.

Then, a modified Poisson regression with generalized estimate equations (GEE) was performed using the identified profiles to predict suicidal behaviors (ideation, planning, and attempted suicide) after adjustment for sociodemographic characteristics. This model allowed us to deduce a prevalence ratio (PR) and its confidence interval. The significance threshold was 5%. To perform this regression, the “geepack” package version 1.3–2 was used.

## 3. Results

### 3.1. Sociodemographic and Behavioral Characteristics of Adolescents

Almost three-quarters of the adolescents included in our study were over the age of 15 (75.1%) and were males (73.0%). The majority of them were in grades 3, 2, 1, or terminale and had relatively high socioeconomic status (65.1%), as shown in Table 2, which summarizes their sociodemographic and behavioral characteristics.

Looking at psychosocial distress, 20.9% of the adolescents suffered from anxiety and 14.2% suffered from loneliness. Concerning physical violence, 22.0% of the adolescents were frequently physically attacked and 23.8% had been involved in a physical fight. The study of socio-environmental factors, represented by the presence or absence of parental support, indicates that 63.6% of the adolescents did not receive enough support. Finally, 7.8% of the adolescents used tobacco (cigarettes or other forms), 44.1% consumed alcohol at least once a month, and 62.0% had insufficient physical activity (inactive).

### 3.2. Suicidality among Adolescents in Benin

A total of 13.8% of the adolescents had suicidal ideation. The prevalence of ideation was higher among adolescents over 15, females, and those with low socioeconomic status, with prevalence equal to 14.6, 18.2, and 17.1%, respectively (Table 3).

The same trends as those observed for ideation were found when looking at planning. Indeed, females, adolescents over the age of 15 at a school level from 3rd grade to terminale, and those with low socioeconomic status were more likely to have already planned suicide, with prevalence of 18.2, 16.8, 16.8, and 18.8%, respectively. The total prevalence of suicidal planning was 15.6%.

In terms of attempted suicide, 15.6% of the adolescents had attempted suicide within the 12 months preceding the survey. The prevalence was highest among those over 15, males and those with low socioeconomic status, equal to 16.7, 16.3, and 18.9%, respectively.

### 3.3. Identified Profiles and Associated Characteristics

Table 4 presents the results obtained from the latent class analysis. Given the BIC values, the distributions into three or four classes were the best. However, if the values of the various criteria, including the adjusted BIC and entropy, were linked and studied, a division into four profiles was chosen. This model, with an entropy equal to 0.76, allowed better distinction between profiles.

Table 5 presents the behavioral and sociodemographic characteristics associated with each profile.

Profile 1, comprising 66.8% of the adolescents in this study, was associated with “good” indicators. This so-called low-risk group was characterized by low alcohol and tobacco consumption, low violence, and low psychological distress compared to the other profiles. Adolescents with high socioeconomic status accounted for the majority of the individuals included in this profile.

The second profile, described as “problems with psychological distress”, comprising 8.6% of the adolescents, was characterized by high proportions of indicators related to psychological distress. In this group, 100.0% of the adolescents experienced loneliness and 64.4% had anxiety. Adolescents over the age of 15 were the most represented (85.6%). This profile is the only one in which the majority of the adolescents had low socioeconomic status.

Adolescents in Profile 3, “problems with violence”, accounted for 15.3% of the total sample. In this group, 100.0% of the individuals had been physically attacked and 35.5% had been involved in physical fights. Males and adolescents with high socioeconomic status represented the majority in this profile.

Finally, Profile 4, described as “problems with alcohol, tobacco and violence”, involved 9.3% of the adolescents and had the highest cumulative risk indicator. In this group, 54.7% of the individuals used tobacco, 96.8% consumed alcohol, 52.2% had been physically attacked, and 78.9% had been involved in physical fights. The proportion of boys in this group was slightly higher than that in the other profiles. High socioeconomic status was also an important feature of this profile.

Physical inactivity and a lack of parental support were relatively common indicators in each profile. However, it should be noted that the adolescents in Profiles 2 and 4 were much more active than those in Profiles 1 and 3. A lack of parental support was a very prominent variable in each profile, with a slight gradual increase in the proportions across the profiles from 1 to 4; Profile 4 had the most adolescents in need of support.

### 3.4. Profiles Obtained and Suicidality

For ideation, planning, and attempts, Table 6 shows that a significant association was found between Profiles 2, 3, and 4 and suicidality compared to Profile 1. For example, the adolescents in Profile 2, “problems with psychological distress,” were more likely than those in Profile 1 to have considered suicide, with a prevalence ratio equal to 1.13 (95% CI = 1.03–1.23), and also more likely to have planned or attempted suicide, with respective prevalence ratios equal to 1.12 (95% CI = 1.04–1.22) and 1.09 (95% CI = 1.01–1.17). The same was observed for the adolescents in Profiles 3 and 4 compared to Profile 1.

## 4. Discussion

Suicide is a social health problem among young people worldwide [25]. Our study aimed to assess the prevalence of suicidality and identify the different behavioral profiles related to lifestyle and their association with suicidality among adolescents in Benin. The results show that approximately one out of five school-going adolescents in Benin reported suicidal ideation, planning, or attempts during the 12 months previous to the study. There were three key findings from this study: First, adolescents over the age of 15, as well as those with low socioeconomic status, were more prone to suicidal behaviors, and women were more prone to suicidal ideation and planning while men were more prone to attempting suicide. Second, four profiles were identified following latent class analysis: low risk, problems with psychological distress, problems with violence, and problems with alcohol, tobacco, and violence. Third, for each suicidal behavior (ideation, planning, and attempts), a significant association was found with Profiles 2, 3, and 4 compared to Profile 1.

### 4.1. Behavioral Profiles Related to Lifestyle and Suicidality

Our study, as it was done in another study in Mauritius for example [26], supports the existence of different profiles of individuals with suicidal behaviors in terms of lifestyle factors.

After the latent class analysis, four profiles were identified among adolescents in Benin. The first profile is described as the low-risk group, among whom low alcohol and tobacco consumption, no physical abuse, and low psychosocial distress were reported. This group comprised more than half of the adolescents in the study (66.8%), and most of them had high socioeconomic status. In fact, a study of adolescents in 34 countries in Europe and America showed that internationally, the higher the income per person, the better the health of adolescents in terms of physical activity, psychological symptoms, and life satisfaction [27]. The second profile, comprising about one in ten adolescents, was characterized by problems with psychosocial distress. In this group, all the adolescents felt loneliness and more than half had anxiety, and this profile mostly comprised subjects over the age of 15. A study by the Psychiatric Service of the Université Libre de Bruxelles showed that adolescents undergo important transformations at the social and individual levels and redefine their social networks, which makes them more fragile and likely to develop mental issues such as depressive symptoms, low self-esteem, anxiety, and stress [28]. Profile 3 was described as a group that had problems with violence and tobacco and alcohol consumption. In this group, representing 15.3% of the population of schooled adolescents, all the subjects had suffered physical attacks and about one in three of them had been involved in physical fights. Profile 4 was characterized by multi-hazard behaviors. This group comprised about one in ten of the surveyed adolescents, and they had problems with alcohol, tobacco, and violence, making this a high-risk group. All the categories of substance use have been shown to be associated with an increased risk of suicide mortality [29]. Lynch et al., in their study in 2020, found that the adjusted odds ratios ranged from 2.0 for patients with only a smoking-related disorder to 11.2 for patients with multiple alcohol, drug, and tobacco-related disorders [29], meaning that the greater the number of health-risk behaviors, the higher the risk of suicide. In this profile, a lack of parental support was the most observed factor leading to a predisposition to suicidal behavior. Macali et al., in their study in 2018, showed that a lack of parental support in adolescence was strongly associated with frequent suicidal thoughts in adulthood [30].

With regard to an association with suicidality, it was observed in our study that adolescents in Profiles 2, 3, and 4 were at significantly higher risk for suicidal-ideation-type behavior. This increased risk was associated with factors such as psychosocial distress, physical attacks, involvement in physical altercations, smoking and alcohol use, physical inactivity, and a lack of parental support. The discovery of an association between these factors and suicidality is not surprising and has already been found in several studies using data from the GSHS survey in Africa [11,26,31,32,33]. Suicidal ideation and planning were found to be also higher for adolescent girls, while there were no significant differences between age and sex in terms of suicide attempts.

Our findings are consistent with those of a 2017 study by Opong et al., who worked on the GHSH 2012 in Ghana. The high prevalence of these suicidal behaviors among adolescent girls could be explained by several biopsychosocial factors, including gender inequality, a tendency for women to internalize their distress more than men, and the increased exposure of women to forms of abuse, particularly childhood sexual abuse, in addition to biological factors such as estrogen levels [34]. Our findings also show that adolescents with low socioeconomic status in Benin were more likely to engage in suicidal behaviors such as ideation, planning, and attempted suicide. This could be explained by the fact that the more children face poverty, the more vulnerable they are to exploitation, violence, abuse, and all forms of discrimination and inequality [35], which can lead to psychosocial depression and, consequently, suicide. Moreover, it has been stated that psychosocial distress is a proxy for suicidal ideation [36]. Thus, in the context of worsening poverty in Benin, where nearly half (43.4%) of children aged 0 to 17 lived in a poor household in 2015 [34], this factor should be seriously considered in strategies to address suicide among adolescents.

### 4.2. Limitations and Strengths

Our conclusions should be interpreted in light of some limitations. First, the main limitation is the type of study. Since this is a cross-sectional study, causal links cannot be established. Second, our study relied on self-reported measures, which may be susceptible to various errors and biases, such as desirability or memory bias. Moreover, the variables used in this study were mostly categorized in only a binary pattern, and only two possible options were given. For example, the measures of anxiety and loneliness did not allow the study of several dimensions, and only two categories (presence/absence) were given. In order to better understand the different possible dimensions of these measures, the GAD-7 scale, which uses a score from 0 to 21 to asses generalized anxiety disorder, or the Revised Child Anxiety and Depression Scale (RCADS) could be used in further studies [37,38]. Finally, the results obtained cannot be extrapolated to the national level, since the data are only about schooled adolescents, in a context where one in three children in Benin was out of school in 2015.

However, our study is the first to take into account the factors in their entirety among adolescents in Benin. Latent class analysis is a robust, person-centered approach to identifying profiles and thus exploring concurrent risk behaviors in adolescents. Additionally, the study was conducted on a large sample with a high response rate of 78%.

## 5. Conclusions

This study shows a relatively high prevalence of suicidal ideation, planning, and attempts among school-going adolescents in Benin, with females more likely to engage in suicidal ideation and planning than males. It shows, for the first time, patterns of health-related lifestyle habits associated with suicidal behaviors among Beninese adolescents. A holistic consideration of these factors should be taken into consideration when designing and developing suicide prevention interventions and strategies in Benin.

## Figures and Tables

**Table 1:** Survey questions and answers used to assess sociodemographic characteristics and health-related lifestyle habits in adolescents aged 11–18 in Benin, 2016.

Variable	Survey Questions	Modalities	Answers
**Psychosocial distress**			
Anxiety	During the past 12 months, how often have you been so worried about something that you could not sleep at night?	Anxiety/Absence of Anxiety	Most of the time or always/never; rarely or sometimes
Loneliness	During the past 12 months, how often have you felt lonely?	Loneliness/Absence of loneliness	Most of the time or always/never; rarely or sometimes
Physical attack	During the past 12 months, how many times were you physically attacked?	Physically attacked/Not physically attacked	At least 1 time/0
Physical fight	During the past 12 months, how many times were you in a physical fight?	Involved in a physical fight/Not involved in a physical fight	At least 1 time/0
**Socio-environmental factors**			
Parental support	During the past 30 days, how often did your parents or guardians check to see if your homework was done?	Parental support/Absence of parental support	Most of the time or always/never; rarely or sometimes
**Health Risk Behaviors**			
Tobacco consumption	During the past 30 days, on how many days did you smoke cigarettes? And during the past 30 days, on how many days did you use any tobacco products other than cigarettes, such as a pipe, rolled tobacco leaves, snuff, or chewing tobacco?	Tobacco consumption/No tobacco consumption	At least 1 day/0 day
Alcohol consumption	During the past 30 days, on how many days did you have at least one drink containing alcohol?	Alcohol consumption/No alcohol consumption	At least 1 day/0 day
Physical activity	During the past 7 days, on how many days were you physically active for a total of at least 60 min per day?	Active/Inactive	More than 60 min for at least 5 days (yes)
**Sociodemographic characteristics**			
Age	How old are you?	≤15/>15	11; 12; 13; 14; 15/16; 17; 18
Sex	What is your sex?	Male/Female	
Grade	In what class are you?	6; 5; 4; 3; 2; 1; Terminale	6th; 5th; 4th; 3rd; 2nd; 1st; Terminal
Socioeconomic status	During the past 30 days, how often did you go hungry because there was not enough food in your home?	High/Low	No or rarely/sometimes; most of the time or always.

**Table 2:** Sociodemographic characteristics and health-related lifestyle habits of adolescents aged 11–18 in Benin, 2016.

	Frequency	Percentage
**Sociodemographic characteristics**		
Age (mean = 16.4 ± 1.6)		
11–15	725	24.9%
>15	1810	75.1%
Missing	1	
Sex		
Male	1366	73.0%
Female	1151	27.0%
Missing	19	
Grade		
6–5–4	924	30.4%
3–2–1-Term	1606	69.6%
Missing	6	
Socioeconomic status		
Low	838	34.9%
High	1687	65.1%
Missing	11	
**Psychosocial distress**		
Anxiety	523	20.9%
Loneliness	360	14.2%
Physically attacked	531	22.0%
Involved in a physical fight	569	23.8%
**Socio-environmental factors**		
Absence of parental support	1602	63.6%
**Health risk Behaviors**		
Tobacco consumption	148	7.8%
Alcohol consumption	961	44.1%
Inactive	1630	62.0%

**Table 3:** Prevalence of suicidality among adolescents aged 11–18 in Benin, 2016.

	Ideation Prevalence [*CI* 95%]	Planification Prevalence [*CI* 95%]	Attempt Prevalence [*CI* 95%]
**Total**	**13.8% [11.8–16.0]**	**15.6% [13.3–18.0]**	**15.6% [13.0–19.0]**
Age			
11–15	11.7% [8.9–15.0]	12.0% [9.2–16.0]	12.3% [9.4–16.0]
>15	14.6% [12.3–17.0]	16.8% [13.9–20.0]	16.7% [13.7–20.0]
Sex			
Male	12.2% [10.3–15.0]	14.6% [12.2–17.0]	16.3% [13.3–20.0]
Female	18.2% [14.5–23.0]	18.2% [14.6–22.0]	13.6% [11.4–16.0]
Grade			
6–5–4	13.1% [8.7–19.0]	13.0% [9.2–18.0]	15.7% [11.7–21.0]
3–2–1-Term	14.2% [11.4–17.0]	16.8% [14.0–20.0]	15.5% [12.6–19.0]
Socioeconomic status			
High	12.2% [9.9–15.0]	13.9% [11.5–17.0]	13.9% [11.4–17.0]
Low	17.1% [13.6–21.0]	18.8% [14.4–24.0]	18.9% [14.3–25.0]

Notes: 95% *CI*: 95% confidence interval.

**Table 4:** Identification of number of profiles obtained among adolescents aged 11–18 in Benin, 2016.

Number of Class	*BIC*	*aBIC*	*cAIC*	*LR*	Class 1	Class 2	Class 3	Class 4	Class 5	Class 6	Entropy
1	21,058.69	21,033.27	21,066.69	734.84	100 %	-	-	-	-	-	-
2	20,781.15	20,727.14	20,798.15	386.84	16.62%	83.38%	-	-	-	-	0.46
3	20,735.52	20,652.91	20,761.52	270.73	11.93%	72.49%	15.59%	-	-	-	0.64
4	20,748.55	20,637.35	20,783.55	213.30	66.76%	9.3%	15.31%	8.63%	-	-	0.76
5	20,794.47	20,654.67	20,838.47	188.74	6.64%	7.63%	7.99%	62.9%	14.83%	-	0.54
6	20,851.31	20,682.92	20,904.31	175.12	62.94%	7.71%	15.27%	3.22%	2.15%	8.71%	0.54

Notes: *BIC*: Bayesian information criterion; *aBIC*: adjusted *BIC*; *cAIC*: consistent Akaike information criterion; *LR*: Likelihood ratio.

**Table 5:** Characteristics of profiles obtained among adolescents aged 11–18 in Benin, 2016.

	Profile 1	Profile 2	Profile 3	Profile 4
**Sociodemographic characteristics**				
Age				
11–15 ans	24.8%	14.4%	32.2%	23.6%
>15 ans	75.2%	85.6%	67.8%	76.4%
Sex				
Male	69.9%	73.3%	77.0%	86.9%
Female	30.1%	26.7%	23.0%	13.1%
Grade				
Grade 6–5–4	29.1%	20.7%	40.9%	32.4%
Grade 3–2–1-Term	70.9%	79.3%	59.1%	67.6%
Socioeconomic status				
High	66.1%	49.5%	64.0%	72.4%
Low	33.9%	50.5%	36.0%	27.6%
**Psychosocial distress**				
Anxiety	16.3%	64.4%	16.2%	21.7%
Loneliness	2.7%	100.0%	10.8%	20.1%
Physically attacked	0.0%	9.3%	100.0%	52.2%
Involved in a physical fight	13.6%	8.8%	35.5%	78.9%
**Socio-environmental factors**				
Absence of parental support	61.2%	63.7%	68.6%	70.9%
**Health risks behaviors**				
Tobacco consumption	1.6%	6.9%	0.0%	54.7%
Alcohol consumption	36.1%	47.2%	37.5%	96.8%
Inactive	68.1%	49.5%	62.1%	36.0%

**Table 6:** Association between suicidality and profiles among adolescents aged 11 to 18 in Benin, 2016.

	Ideation PR [CI 95%]	Planification PR [CI 95%]	Attempt PR [CI 95%]
**Profile (reference: Profile 1, low risk group)**			
Profile 2 (problems with psychological distress)	1.13 [1.03–1.23] *	1.12 [1.04–1.22] **	1.09 [1.01–1.17] *
Profile 3 (problems with violence)	1.06 [1.00–1.13] *	1.11 [1.06–1.16] ***	1.08 [1.03–1.13] ***
Profile 4 (problems with alcohol, tobacco, and violence)	1.09 [1.04–1.15] **	1.12 [1.06–1.18] ***	1.07 [1.01–1.14] *
**Sociodemographic characteristics**			
Sex (reference: Male)			
Female	1.07 [1.04–1.11] ***	1.05 [1.02–1.09] **	0.99 [0.97–1.01]
Age (reference: 11–15 ans)			
>15 ans	1.03 [1.00–1.07]	1.05 [1.01–1.09] *	1.04 [1.00–1.08]
Socioeconomic status (reference: low)			
High socioeconomic status	0.95 [0.92–0.99] **	0.96 [0.92–1.00] *	0.96 [0.92–0.99] *

Notes: *PR*: prevalence ratio; 95% *CI*: 95% confidence interval; *: *p* < 0.05; **: *p* < 0.01; ***: *p* < 0.001.

## Data Availability

Data for this study were obtained from the World Health Organization (WHO) website, is freely available online, and can be downloaded (https://extranet.who.int/ncdsmicrodata/index.php/catalog/627/get_microdata accessed on 25 June 2021).
